# Development of a rapid and sensitive analytical system for *Pseudomonas aeruginosa* based on reverse transcription quantitative PCR targeting of rRNA molecules

**DOI:** 10.1080/22221751.2021.1906164

**Published:** 2021-04-02

**Authors:** Mai Niikura, Satomi Atobe, Akira Takahashi, Yukiko Kado, Takuya Sugimoto, Hirokazu Tsuji, Kentaro Shimizu, Hiroshi Ogura, Takashi Asahara

**Affiliations:** a Yakult Central Institute, Yakult Honsha Co., Ltd., Kunitachi, Tokyo, Japan; b Department of Traumatology and Acute Critical Medicine, Osaka University Graduate School of Medicine, Suita, Osaka, Japan

**Keywords:** *Pseudomonas aeruginosa*, infection, RT-qPCR, blood, feces, ICU patients, antimicrobial resistance, microbiota

## Abstract

For *Pseudomonas aeruginosa* (PA), infection control and appropriate antimicrobial treatment have become important issues. Diagnosis is critical in managing PA infection, but conventional methods are not highly accurate or rapid. We developed a new PA quantification system based on 23S rRNA-targeted reverse transcription quantitative PCR (RT-qPCR). We confirmed that RT-qPCR can quantify PA directly from clinical samples quickly (within 6 h) and with high sensitivity (blood, 1 cell/mL; stool, 100 cells/g) and without cross-reaction. Also, under antibiotic treatment, PA viable counts detected by this system correlated well with the inflammatory response of infected Caco-2 cells compared to other methods such as culturing and qPCR. Next, we utilized this system on fecal samples collected from 65 septic ICU patients and 44 healthy volunteers to identify ICU infection status. We confirmed that the PA detection ratio in ICU patients was significantly higher than that in healthy volunteers (49.2% vs. 13.6%, *P* < 0.05). Additionally, we monitored drug-resistant PA in 4 ICU patients by this system. The trends in PA counts accurately reflected various treatment backgrounds such as antibiotic use and mechanical ventilator use. Our results suggest that this RT-qPCR system is beneficial for the early diagnosis and evaluation of appropriate antibacterial treatment and may be a useful tool in combating PA infection.

## Introduction

*Pseudomonas aeruginosa* (PA), a ubiquitous bacterial species found in a wide variety of environmental settings, causes nosocomial infection [1] that can be critical and inflict high mortality [2].

Recently, the prevalence of multidrug-resistant PA (MDRP), i.e. strains that are resistant to antibiotics such as fluoroquinolones, carbapenems, and aminoglycosides, has become a serious problem [3]. Infection with MDRP can limit antibiotic therapy, resulting in inappropriate empirical therapy or delays in initiating appropriate therapy, which can lead to high mortality [4,5]. Thus, in order to select appropriate antibacterial agents for treating PA infection, it is necessary to rapidly and accurately identify PA, including MDRP strains, in clinical specimens.

Generally, bacterial culture methods are the gold standard for detecting PA; however, culture methods have several limitations: (i) they take time (2–3 days) [6]; (ii) they only detect culturable pathogens that have the ability to form colonies [7]; and (iii) they can underestimate the number of pathogens due to selection bias induced by administered antibiotics that are present in human blood [8]. Accordingly, culture methods can produce false-negative results. Culture-negative sepsis is commonly observed in ICU patients: previous studies have shown negative blood cultures in 30% of septic shock patient [9]. It has also been reported that blood cultures from febrile neutropenic patients can identify the pathogens in only 17%–42% of cases [10,11]. Thus, to overcome the problems associated with bacterial culture, we adopted Yakult Intestinal Flora-SCAN (YIF-SCAN®), a highly sensitive and rapid system based on reverse transcription quantitative PCR (RT-qPCR) [12–14]. YIF-SCAN® targets rRNA molecules that exist in bacteria abundantly (approximately 10^4^ copies per actively growing cell), and the sensitivity of RT-qPCR is 100 times higher than that of qPCR assays that target rRNA genes (more than 10 copies/bacterial genome) [12]. Therefore, RT-qPCR is a more sensitive assay for detecting occult bacteria. Also, it requires only 6 h for the quantification of bacteria and detects only live bacteria from specimens [14,15].

In the present study, in order to establish a useful tool to combat PA infection, we developed a new PA quantification system based on 23S rRNA-targeted RT-qPCR. We assessed the performance and potential impact of RT-qPCR on antibiotic therapy administered to ICU patients.

## Materials and methods

### Ethics statement

Human samples were anonymously coded in accordance with local ethical guidelines (as stipulated by the Declaration of Helsinki). Written informed consent was obtained from the patients and healthy volunteers. This study was approved by the Ethical Review Board of Osaka University (approval number: 09184-5) and Yakult Central Institute (FY2017-025, FY2018-028).

### Strains and culture conditions

The bacterial strains used are listed in Table 1. Each strain was grown under the conditions listed in Table S1. The colony forming units (CFU) of PA were determined by culturing aerobically on trypticase soy agar (TSA, Beckton Dickinson, Franklin Lakes, NJ, USA) or the newly developed selective medium, NCC agar (Supplemental information), at 37°C for 24 h.

**Table 1.** Specificity tests with the newly designed primer set.

Taxon	Strain	Reaction^a^
*Pseudomonas aeruginosa*	ATCC 10145^T^	+
	ATCC 9027	+
	ATCC 15442	+
	JCM 2776	+
	JCM 5961	+
	DSM 6195	+
*Pseudomonas alcaligenes*	ATCC 14909^T^	−
*Pseudomonas fluorescens*	ATCC 13525^T^	−
	ATCC 15916	−
*Pseudomonas brenneri*	ATCC 49642	−
*Pseudomonas luteola*	ATCC 43273^T^	−
*Pseudomonas oryzihabitans*	JCM 2952^T^	−
	JCM 3843	−
*Pseudomonas pseudoalcaligenes*	JCM 5968^T^	−
*Pseudomonas putida*	ATCC 12633^T^	−
	ATCC 49128	−
*Pseudomonas stutzeri*	ATCC 17588^T^	−
*Pseudomonas tolaasii*	ATCC 33618^T^	−
*Bacteroides vulgatus*	ATCC 8482^T^	−
*Bifidobacterium adolescentis*	ATCC 15703^T^	−
*Blautia producta*	JCM 1471^T^	−
*Clostridium difficile*	DSM 1296^T^	−
*Clostridium perfringens*	JCM 1290^T^	−
*Collinsella aerofaciens*	DSM 3979^T^	−
*Enterococcus faecalis*	ATCC 19433^T^	−
*Escherichia coli*	ATCC 11775^T^	−
*Faecalibacterium prausnitzii*	ATCC 27768^T^	−
*Prevotella melaninogenica*	ATCC 25845^T^	−
*Lactobacillus brevis*	ATCC 14869^T^	−
*Lactobacillus casei*	ATCC 334^T^	−
*Lactobacillus fermentum*	ATCC 14931^T^	−
*Lactobacillus fructivorans*	ATCC 8288^T^	−
*Lactobacillus gasseri*	DSM 20243^T^	−
*Lactobacillus plantarum*	ATCC 14917^T^	−
*Lactobacillus reuteri*	JCM 1112^T^	−
*Lactobacillus ruminis*	JCM 1152^T^	−
*Lactobacillus sakei*	ATCC 15521^T^	−
*Staphylococcus aureus*	ATCC 12600^T^	−
*Streptococcus mutans*	ATCC 27175^T^	−

^a^ The specificity assay with the s-Pa-F/s-Pa-R primer set was investigated by using RNA extracts corresponding to 10^5^ cells from each strain described.

+: when it was more than that of 10^4^ standard cells; −: when it was less than that of 10^1^ standard cells. The amplified signal was also defined as negative (−) when the corresponding melting curve had a peak different from that of the standard strain.

### Development of 23S rRNA gene-targeted primers specific to P. aeruginosa

First, we newly sequenced the 23S rRNA genes of 6 strains of PA and 8 species related to PA. Sequence analysis was performed using a previously described method [16]. Each primer set used in the amplification is described in Table S2. The 23S rRNA gene sequences were deposited in the DNA Data Bank of Japan (DDBJ) nucleotide sequence database under accession numbers listed in Table S3. Next, a multiple alignment of the 6 PA strains and 8 related species was performed with the CLUSTAL_X program [17] using the obtained 23S rRNA gene sequences. After a comparison of the sequences *in silico*, target sites for PA species-specific detection were identified, and a primer set, s-Pa-F (5′-GTCGTCTTTTAGATGACGAAGTGG-3′) and s-Pa-R (5′-TGGTATCTTCGACCAGCCAGA-3′), was newly constructed. The potential target sites for specific detection were identified (Table S4). The specificity of the designed primer pair was then confirmed by submitting the sequences to the BLAST program of the National Center for Biotechnology Information (NCBI) (http://blast.ncbi.nlm.nih.gov/Blast.cgi).

### Total RNA extraction and RT-qPCR

For RNA stabilization, 2 volumes of RNAprotect Bacteria Reagent (QIAGEN, Hilden, Germany) were added to samples. After being kept for 10 min at room temperature, the samples were centrifuged at 13,000 × *g* for 10 min. The supernatant was discarded, and the pellet was stored at −80°C until used for RNA extraction. RNA extraction and RT-qPCR using a QIAGEN OneStep RT-PCR kit were performed as described elsewhere [12]. In the case of bacterial cultures or blood samples, assays were performed in 96-well optical plates (WATSON, Tokyo, Japan) using a 7500 Real-Time PCR System (Thermo Fisher, Waltham, MA, USA). For fecal samples, 384-well optical plates (Life Technologies, Carlsbad, CA, USA) with a QuantStudio^TM^ 12K Flex Real-Time PCR System (Thermo Fisher) were used. The reaction mixture was incubated at 50°C for 30 min for reverse transcription. In either case, the continuous amplification program consisted of one cycle at 95°C for 15 min and 45 cycles at 94°C for 20 s, 60°C for 20 s, and 72°C for 35 s. Standard curves were generated using the threshold cycle (*C_q_*) values and the corresponding cell counts, which were determined with DAPI staining [18].

### DNA extraction and qPCR

Samples were centrifuged at 13,000 × *g* for 10 min, and the supernatant was discarded. The pellet was stored at −80°C until used for DNA extraction. DNA extraction was performed using a previously described method [19]. qPCR was carried out using a QIAGEN OneStep RT–PCR kit. Each reaction mixture contained the same components as those for RT-qPCR, except for the replacement of template RNA with the same amount of template DNA. The reaction mixture was incubated at 95°C for 15 min and 45 cycles at 94°C for 20 s, 60°C for 20 s, and 72°C for 35 s. The subsequent procedures were the same as those for RT-qPCR.

### Determination of primer specificity

Total RNA fractions extracted from the bacterial cells of each strain (shown in Table 1) at a dose corresponding to 10^5^ cells were assessed by RT-qPCR using the primer set of s-Pa-F and s-Pa-R.

### Determination of RT-qPCR sensitivity

Total RNA and DNA fractions of the 6 strains of PA were extracted from culture samples, and bacterial counts were determined with DAPI staining. Serial RNA and DNA dilutions corresponding to bacterial counts ranging from 10^−2^ to 10^4^ cells were assessed by RT-qPCR and qPCR assays, respectively.

### Quantification of P. aeruginosa spiked to human blood and human feces by RT-qPCR and culture methods

Commercially available human blood type A (Kohjin Bio Co., Saitama, Japan) and fecal samples collected from three healthy adult volunteers that had been confirmed in advance not to include PA were used. As for fecal samples, weighed samples were suspended in 9 volumes of trypticase soy broth (TSB, Beckton Dickinson). PA ATCC 10145^T^ was serially diluted with TSB and then spiked to make final concentrations ranging from 10^0^ to 10^4^ cells/mL of blood and 10^2^ to 10^7^ cells/g of feces. For RT-qPCR assays, 1 mL of the blood sample and 120 μL (4 mg equivalent feces) of the fecal homogenate was used for RNA extraction. For the culture method, human blood samples (1 mL) or fecal homogenates (100 μL of each appropriate dilution series) spiked with PA were cultured on NCC agar plates.

### Monitoring of MDRP exposed to antibiotics by three different methods

MDRP ATCC BAA-2108^TM^ adjusted to a final concentration of 10^6^ CFU/mL was cultured in Dulbecco’s modified Eagle’s medium (DMEM, Thermo Fisher) containing 10% fetal bovine serum (FBS, Thermo Fisher), 1% MEM nonessential amino acids (Thermo Fisher), 5 μg/mL colistin sulfate salt (Sigma-Aldrich Co., St. Louis, MO, USA), and 100 μg/mL doripenem hydrate (SHIONOGI & CO., LTD., Osaka, Japan) at 37°C with shaking (140 rpm). After the start of culture, 10 mL of the culture solution was periodically sampled and washed by centrifugation at 13,000 × *g* for 10 min, and the supernatant was discarded. The precipitated pellets were resuspended in 10 mL of antibiotic-free and serum-free DMEM. Then, the culture solution was distributed for RNA extraction (1 mL), DNA extraction (1 mL), culturing for CFU counts (1 mL), cell infection (2 mL), and acquisition of fluorescent images (3 mL). Nucleic acid extraction, RT-qPCR, and qPCR were performed as described above. For culturing for CFU counts, 1 mL of the appropriate dilution series was cultured on TSA plates.

### Quantification of inflammatory response in infected cells

Caco-2 cells (86010202) were plated on 6-well plates (Costar, Corning, NY, USA) at a density of 3.0 × 10^5^ cells/well and used in the infection assays. For cell infection, 2 mL of antibiotic-treated MDRP suspension was applied to the cells, and cells were incubated at 37°C with 5% CO_2_ in air for 6 h. The bacterial proinflammatory effect on Caco-2 cells was assessed. After a 6-h incubation, 2 mL of the cell culture medium was sampled and centrifuged at 2,300 × *g* for 5 min. The supernatant was stored at −80°C until IL-8 production was measured. IL-8 production was assayed using an ELISA Quantikine kit (BioLegend). Total RNA was extracted from infected Caco-2 cells using TRIzol reagent (Thermo Fisher). IL-8 [20] and GAPDH [21] gene expression analyses were performed by RT-qPCR.

### Observation and acquisition of fluorescent images

For acquisition of fluorescent images, the bacterial suspension was centrifuged at 13,000 × *g* for 10 min and then concentrated 100-fold. Fluorescence *in situ* hybridization (FISH) analyses were performed as described previously with minor modifications [22].

### Monitoring of drug-resistant P. aeruginosa in clinical samples and fecal microbiota analysis

For the analysis of clinical fecal samples, we used total RNA collected in a previous study [23], which were stored at −80°C until used in this study. In the previous study, fecal samples were acquired from 65 patients who were more than 16 years old and had been placed on a ventilator within 3 days after admission to the ICU, and who were diagnosed as having sepsis in the Department of Traumatology and Acute Critical Medicine, Osaka University Medical School, and Osaka General Medical Center during the period from November 2011 to September 2016. Also, we collected fecal samples from 44 healthy adults and prepared total RNA. Then, three serial dilutions of each extracted RNA sample were used for RT-qPCR using the primer set of s-Pa-F and s-Pa-R. Furthermore, when monitoring drug-resistant PA in fecal samples of 4 ICU patients by RT-qPCR, drug-resistance gene-specific primers (*bla*_IMP_ [24], *ampC* [25], and *mexA* [26]) were also used. The fecal microbiota compositions of the 4 ICU patients were analyzed using the YIF-SCAN® system as described previously [12].

### Statistical analysis

Statistical analyses were performed using IBM SPSS Statistics Desktop version 22.0 software (IBM Japan Ltd.). *P* values <0.05 were considered statistically significant. Data are presented as mean and standard deviation of representative experiments.

## Results

### Specificity tests with the newly designed primer set

We evaluated the specificity of the newly designed primer set. Among 39 strains, including 6 strains of PA, 12 species related to PA, and 21 strains of enteric bacteria or causative agents of human intestinal tract infectious disease, the primer set reacted only with the 6 strains of PA and showed no reaction with the other 33 bacterial strains investigated (Table 1). This confirmed that the primer set targeting PA is specific for the target strains.

### Quantification of P. aeruginosa by RT-qPCR and comparison of sensitivity with qPCR

**Figure 1.**
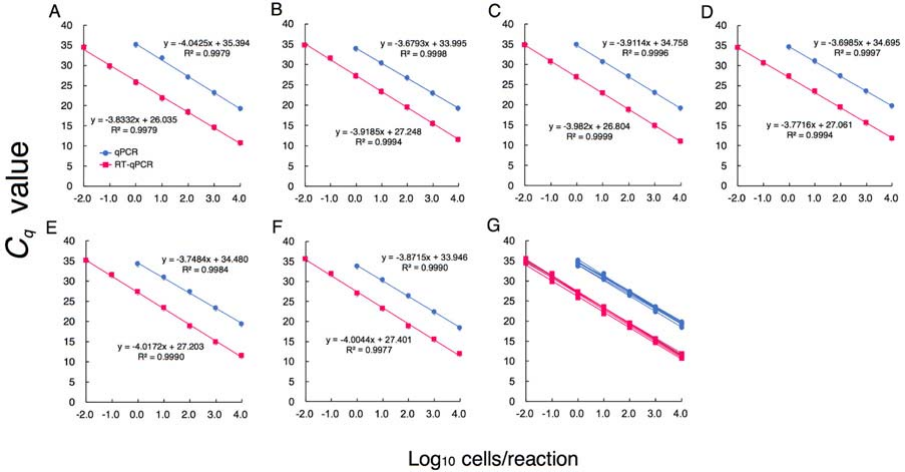
Quantification of *P. aeruginosa* by RT-qPCR and comparison of sensitivity with qPCR. PA ATCC 10145^T^ (A), ATCC 9027 (B), ATCC 15442 (C), JCM 2776 (D), JCM 5961 (E), and DSM 6195 (F) were cultured separately. RNA and DNA were extracted from cultured samples in the early stationary phase. Bacterial counts were determined microscopically with DAPI staining. Based on bacterial counts, 10-fold serial dilutions of RNA or DNA from 10^−2^ to 10^4^ cells were assessed. The *C_q_* values obtained were plotted against the log_10_ number of bacterial cells subjected to each reaction (*n* = 3). Values are the mean ± SD. (G) The parameters from the linear regression analyses for the 6 different PA strains.

The counts of PA in pure cultures obtained by DAPI staining and *C_q_* values obtained by RT-qPCR showed a good correlation over the range of RNA amounts corresponding to 10^−2^–10^4^ bacterial cells/reaction (Figure 1A–F). The counts of PA also correlated well with the *C_q_* values obtained by qPCR; however, the detection limit was 10^0^ cells/reaction (Figure 1A–F). These results indicated that RT-qPCR is approximately 100-fold more sensitive than qPCR. The parameters from the linear regression analyses for the 6 different PA strains were highly similar, indicating they had the same quantitative performance (Figure 1G).

### Quantification of P. aeruginosa spiked to human blood or feces using RT-qPCR and culture methods

**Figure 2.**
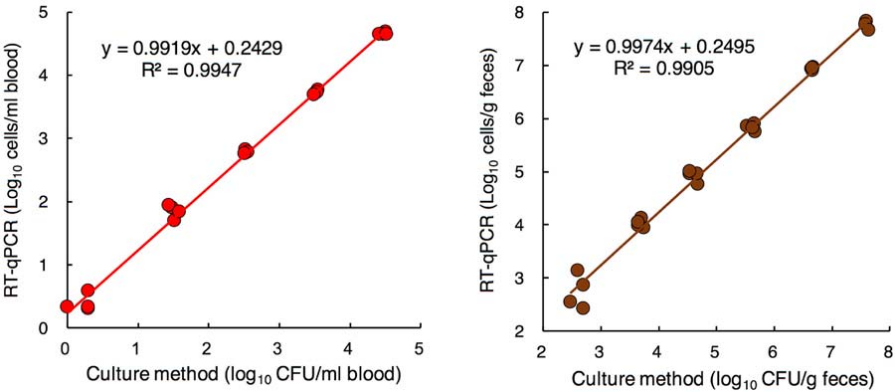
Quantification of *P. aeruginosa* spiked to human blood or feces using RT-qPCR and culture methods. Commercially available human peripheral blood (A) and fecal samples collected from 3 individuals (B) were spiked with serial dilutions of PA ATCC 10145^T^ at final concentrations ranging from 10^0^ to 10^4^ cells/mL of blood and 10^2^ to 10^7^ cells/g of feces. The total counts of PA were determined by RT-qPCR and the culture method (*n* = 4). For the culture method, an appropriate dilution series of each blood sample was cultured on NCC agar plates (selective medium).

The spiked bacterial counts could be detected by RT-qPCR at concentrations as low as 10^0.3^ cells/mL of blood and 10^2.4^ cells/g of feces. Also, the bacterial counts detected by the culture method and those determined by RT-qPCR showed a good correlation (Figure 2A and B).

### Comparison of detection methods in generating time-kill curves for MDRP exposed to antimicrobials

**Figure 3.**
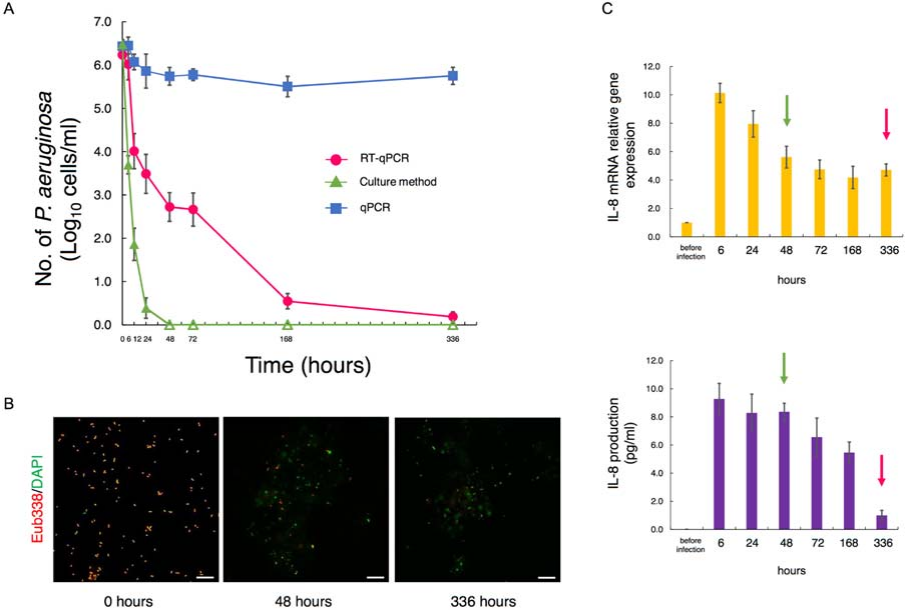
Comparison of detection methods in generating time-kill curves for MDRP exposed to antimicrobials. (A) Time-kill curves for PA ATCC BAA-2108^TM^ exposed to colistin and doripenem combinations (*n* = 4). Bacterial counts in the culture solutions sampled over time were determined by RT-qPCR, the culture method, and qPCR. For RT-qPCR and qPCR, the primer set targeting PA-specific 23S rRNA was used. For the culture method, the appropriate dilution series were cultured on TSA plates. When the bacterial count was not detected (<1 cell/mL), the circles were rendered as open. (B) Parts of the culture solutions were subjected to measurement of bacterial counts and assayed by FISH with FITC-labeled Eub338. Green and red images of PA were obtained by DAPI and FISH, respectively. Scale bars: 10 μm. (C) After Caco-2 cells were infected with a part of the culture solution used for determination of bacterial counts, relative amounts of IL-8-encoding transcripts were measured by RT-qPCR. Expression was normalized to GAPDH, and the relative gene expression values were compared to those of uninfected cells (*n* = 4). The production of IL-8 was measured by ELISA (*n* = 4). The red and green arrows indicate the point at which PA was thought to have disappeared by RT-qPCR and the culture method, respectively. Data indicate the mean ± SD.

Changes over time in the counts of antibiotic-treated MDRP differed greatly depending on the measurement method (Figure 3A). The counts detected with RT-qPCR gradually decreased until 336 h after antibiotic treatment. In contrast, the counts detected with qPCR showed almost no change. The counts detected with the culture method decreased rapidly after antibiotic treatment and were no longer detected 48 h after treatment.

When the PA solution was tested with FISH, a large number of viable bacteria were observed at the start of antibiotic treatment; however, the number of bacteria dramatically decreased, and almost no viable bacteria could be observed after 336 h (Figure 3B).

PA solutions sampled to measure bacterial counts were also used for infection of Caco-2 cells. The relationship between the inflammatory response and the PA counts obtained with each measurement method was evaluated. When Caco-2 cells were infected with 6-h antibiotic-treated PA, the IL-8 gene expression and production levels in cells increased more than 9-fold compared to before infection. Even at 48 h after antibiotic treatment, when PA was no longer detectable by culturing (Figure 3C, green arrows), the IL-8 gene expression and secretion levels were very high. At 336 h, the point when no decrease in cell counts was seen with qPCR, but most PA were thought to have disappeared by RT-qPCR (Figure 3C, red arrows), IL-8 gene expression and production had decreased remarkably. Only the trend in the PA count as monitored by RT-qPCR was similar with the level of Caco-2 cell IL-8 production (Figure 3C).

### Quantification of P. aeruginosa in fecal samples of ICU patients and healthy volunteers

To examine whether RT-qPCR can be applied to clinical samples and to identify the ICU infection status, RT-qPCR analysis was performed using fecal samples collected from 65 ICU patients (Table 2). PA was detected 49.2% at an average count of 10^5.48±1.90^ cells/g of feces. In contrast, in 44 healthy volunteer samples, 13.6% were positive for PA and the average count was 10^4.38±1.21^ cells/g of feces (Table 2). The detection ratio in ICU patients was significantly higher than that in healthy volunteers (*P *< 0.05). As for bacterial counts, while some patients showed high levels of PA in the feces (10^8^ cells/g of feces), some patients had very low levels (10^2^ cells/g of feces) as observed in healthy volunteers.

**Table 2.** Quantification of *P. aeruginosa* in fecal samples of ICU patients and healthy volunteers.

Groups	*n* (by gender)^a^	Age range (year, mean ± SD)	Bacterial counts (Detection range)^b^	Detection ratio
ICU patients (*n* = 65)	65 (M 44, F 21)	20–97 (61 ± 19)	10^5.48±1.90^ (10^2.32^–10^8.88^)	49.2% (32/65)*
Healthy adults (*n* = 44)	44 (M 26, F 18)	23–59 (39 ± 11)	10^4.38±1.21^ (10^3.12^–10^5.98^)	13.6% (6/44)

^a^ M, male; F, female.

^b^ Cells/g of feces, data are expressed as the mean ± SD.

*P < 0.05: Fisher’s exact test was used to compare the detection rate of PA between 2 groups. The Mann-Whitney *U* test (2-tailed) was used to compare the average PA counts between 2 groups.

Detection limit: <10^2.3^ (cells/g of feces)

### Monitoring of drug-resistant P. aeruginosa in clinical samples

**Figure 4.**
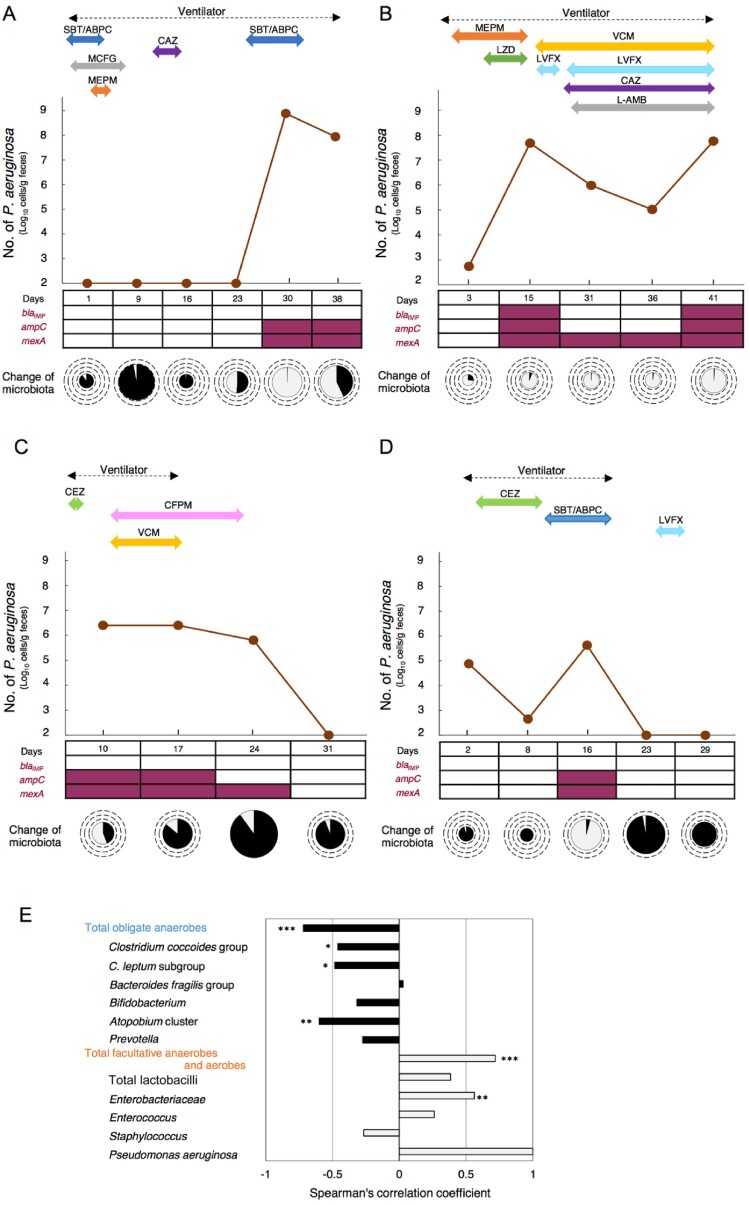
Monitoring of drug-resistant *P. aeruginosa* by RT-qPCR and clinical treatment. RT-qPCR quantification of PA (brown) and simultaneous detection of drug resistance genes (*bla*_IMP_, *ampC*, *mexA*) using drug-resistance gene amplification primers (+, red-square; −, white-square) in fecal samples from 4 ICU patients (A–D). The abscissa of each graph represents the days of hospital stay. The microbiota composition was analyzed using the YIF-SCAN® version of a 16S and 23S rRNA-targeted RT-qPCR system. The size of the pie charts shows the total bacterial counts (circles indicate 10^11^, 10^10^, 10^9^, 10^8^, and 10^7^ cells/g feces in this order from the outside to the inside), and the colour of the pie charts shows the percentage of obligate anaerobes (black) and facultative anaerobes and aerobes (light gray). The duration of ventilator use is indicated by a broken black line, and the duration of antibiotic use during the hospital stay is indicated by the various coloured solid lines. (E) Association between PA counts and fecal occupation rate of representative microorganisms. Spearman's rank correlation coefficient was applied to the dataset. *P < 0.05, **P < 0.01, ***P < 0.001. SBT/ABPC, sulbactam/ampicillin; MCFG, micafungin; MEPM, meropenem; LZD, linezolid; VCM, vancomycin; LVFX, levofloxacin; CEZ, cefazolin; CAZ, ceftazidime; CFPM, cefepime; L-AMB, amphotericin B.

When PA counts in the feces of ICU patients undergoing antibiotic treatment were measured with RT-qPCR, we could monitor abnormal proliferation and excessive decreases of PA in the intestines (Figure 4A–D). Monitoring of drug-resistant PA was also possible with the combined use of drug-resistance gene amplification primers and the PA-specific primers (Figure 4A–D). In the 4 patients in whom PA was detected from the feces, a drug efflux pump gene (*mexA*) and the AmpC β-lactamase gene (*ampC*) were detected from the feces of all patients (Figure 4A–D). From Patient B, who was receiving meropenem (a carbapenem antibiotic), a metallo-beta-lactamase (MBL)-encoding gene (*bla*_IMP_) was detected (Figure 4B).

The trends in PA counts in the feces of ICU patients monitored by RT-qPCR were related to the administration of antibiotics, the use of an artificial ventilator, and the composition of intestinal microbiota (Figure 4 and Table S5). In Patient A, explosive proliferation of PA in the intestines was induced at the time (Day 30) when obligate anaerobe groups rapidly decreased with the administration of antibiotics (Figure 4A). Similar trends were seen in Patient B (Day 15, 41) and Patient D (Day 16) (Figure 4B and D). In contrast, when the administration of antibiotics was ceased, the PA count decreased (Patient A: Day 38, Patient C: Day 31, Patient D: Day 23) (Figure 4A, C and D). In two patients taken off of artificial ventilators, intestinal counts of drug-resistant PA decreased to below the detection limit at the time when the counts of the most prevalent obligate anaerobes recovered after ventilator withdrawal (Patient C: Day 31, D: Day 23) (Figure 4C and D).

Negative correlations were seen between the PA counts and the proportion of total obligate anaerobes (Figure 4E). In contrast, positive correlations were seen between PA counts and the proportions of total facultative anaerobes and aerobes (Figure 4E).

## Discussion

In this study, we developed a new PA quantification system based on 23S rRNA-targeted RT-qPCR. Using the RT-qPCR method, PA was detected from as little as 1 cell/mL of blood and 10^2^ cells/g of feces, with high specificity and quantifiability. When this RT-qPCR method was applied to fecal samples from ICU patients, it demonstrated that PA had colonized some patients at very low levels in the intestine (10^2^ cells/g of feces). RT-qPCR was shown to be useful for accurately determining the infection status or screening of patients who carry minute amounts of PA, which are easy to miss with conventional methods.

The results of microorganism tests such as culturing and qPCR and patients’ clinical conditions do not necessarily coincide [9–11]. In our experimental system using PA-infected cells, the counts of antibiotic-treated PA by RT-qPCR correlated well with the inflammatory response of infected cells. As RT-qPCR targets rRNA that is rapidly degraded in dead cells [12], it can measure only live bacteria that are highly associated with infectivity, inflammation induction, and pathogenicity. In contrast, qPCR targets DNA that continues to remain even in dead bacteria. By DAPI staining, which targets DNA, we observed that fluorescence microscopy images remained fairly constant even at the death phase, so dead PA continued to be observed after antibiotic treatment (Figure 3B). Given this, the high number of PA detected by qPCR, despite the significant reduction in the inflammatory response of the cells, is thought to be due to the detection of both viable and dead cells. The discrepancy between bacterial count results with culture methods and the inflammatory response of infected cells is thought to be due mainly to a decrease in the ability of PA to form colonies in a viable but non-culturable state [7] under antibiotic treatment. From the above, since RT-qPCR can accurately detect PA even during the use of antibiotics, RT-qPCR is considered to be very useful in choosing appropriate antibiotics and determining the timing of treatment.

Previous reports have indicated that more than 70% of ICU patients receive antimicrobial treatment and are therefore susceptible to the development of drug-resistant PA infection [27]. In this study, we demonstrated that intestinal infections of drug-resistant PA in ICU patients can be quickly detected using RT-qPCR. It has been reported that meropenem is associated with the risk of resistance emergence [28]. In Patient B, who had received meropenem for more than 2 weeks, an elevated fecal PA count was observed, and upregulation of the MBL-encoding gene was detected. About 80% of MDRP have been reported to be MBL-positive isolates [29]. Furthermore, PA can actually proliferate in the intestines during the administration of levofloxacin [30]. Therefore, there is a high likelihood that the bacterium detected in the feces was MDRP. This indicates that the RT-qPCR method may also be applicable for rapid and highly sensitive monitoring of such MDRP.

The PA counts with RT-qPCR closely reflected the treatment background of ICU patients. Specifically, the induction of abnormal proliferation of PA in the intestines was observed with the long-term use of various types of antibiotics. It has been shown that the eradication of PA is actually facilitated by discontinuing the administration of sulbactam/ampicillin, which is not active against PA [31], or broad spectrum cephem antibiotics (cefepime), for which drug-resistant PA has low sensitivity [32]. The eradication of PA in the intestines was also observed after the withdrawal of mechanical ventilator use. Thus, we demonstrated that the timely monitoring of fecal PA accompanying medical interventions is possible with this system.

In this study, abnormal proliferation of PA in the intestines occurred in ICU patients with decreased obligate anaerobes in the intestines after antibiotic administration, and a negative correlation was observed between obligate anaerobe counts and PA. In ICU patients, disruption of the gut microbiota has been reported to be a high-risk factor for carbapenem-resistant PA acquisition [33] and mortality [34]. Also, Robak et al. showed that microbiota-dependent IgA production is required for antibacterial immunity during acute bacterial pneumonia [35]. These findings indicate the importance of intestinal microbiota in ICU patients. Since both intestinal dysbiosis and PA infection dynamics can be understood simultaneously, the RT-qPCR method may be a very effective means of controlling the risk of infection in patients.

Our study has several limitations. First, while we attempted to monitor drug-resistant PA using 3 drug-resistance gene primers, PA has various drug-resistance mechanisms. In the future, we anticipate that definitive diagnosis and monitoring of MDRP will also be possible by generating other kinds of resistance gene detection primers. Second, horizontal transfer of the *bla*_IMP_ gene has been reported; thus, the possibility that other carbapenem-resistant pathogens such as *Enterobacteriaceae* or *Acinetobacter baumannii* had the *bla*_IMP_ gene cannot be ruled out. It will also be possible to estimate gene origins by preparing other pathogen-specific primers and monitoring these pathogens. Third, in the monitoring of drug-resistant PA in the feces of ICU patients, the sample size was small. Further studies with larger sample sizes are necessary to clarify the relationship between the fluctuations of PA detected by RT-qPCR and the clinical course. Fourth, although this new method enables very sensitive detection compared to the conventional method, it still cannot quantify the amount of PA below the detection limit (∼10^2^ cells/g) for feces.

In summary, we demonstrated the potential of a new RT-qPCR method as an effective tool for controlling nosocomial infections of PA as well as for judging the efficacy of antibiotic treatment and determining whether to continue or withdraw other medical actions.

## Supplementary Material

20210317_Supplemental-Material_TEMI-2021-0107-R1_editable.docx

## Data Availability

The 23S rRNA gene sequences deposited in the DDBJ (http://www.ddbj.nig.ac.jp/) nucleotide sequence database are listed in Table S3.
