# Supplementary material for: Development of a rapid and sensitive analytical system for Pseudomonas aeruginosa based on reverse transcription quantitative PCR targeting of rRNA molecules
Source: Emerg Microbes Infect. 2021 Apr 2;10(1):677–86. doi: 10.1080/22221751.2021.1906164 (PMC8023615; doi:10.1080/22221751.2021.1906164)
Supplement: 20210317_Supplemental-Material_TEMI-2021-0107-R1_editable.docx [file TEMI_A_1906164_SM9775.docx]

**Supplementary materials**

***NCC agar plates***

NCC agar is a newly developed selective medium for the detection of *P. aeruginosa* in this study, and contains NAC (nalidixic acid, cetrimide) medium (Nissui Pharmaceutical, Tokyo, Japan) and TSA at a ratio of 1:20, 0.5 µg/ml ceftizoxime sodium (Chem-Impex International, Inc., Wood Dale, IL, USA), and 64 µg/ml cephalothin sodium crystalline (Sigma-Aldrich Co., St. Louis, MO, USA).

**Table S1. Strain and culture conditions**

| Taxon | Strain | Medium | Temperature, periods | Gas phase |
| --- | --- | --- | --- | --- |
| *Pseudomonas aeruginosa* | ATCC 10145^T^ | BHI broth ^A)^ | 37 ℃, 18 hours | aerobically |
|  |  | TS broth ^B)^ | 37 ℃, 18 hours | aerobically |
|  | ATCC 9027 | BHI broth | 37 ℃, 22 hours | aerobically |
|  | ATCC 15442 | BHI broth | 30 ℃, 22 hours | aerobically |
|  | JCM 2776 | BHI broth | 30 ℃, 22 hours | aerobically |
|  | JCM 5961 | BHI broth | 37 ℃, 22 hours | aerobically |
|  | DSM 6195 | BHI broth | 30 ℃, 22 hours | aerobically |
|  | ATCC BAA-2108^TM^ | TS broth | 37 ℃, 18 hours | aerobically |
| *Pseudomonas alcaligenes* | ATCC 14909^T^ | BHI broth | 30 ℃, 22 hours | aerobically |
| *Pseudomonas fluorescens* | ATCC 13525^T^ | Nutrient broth ^C)^ | 30 ℃, 26 hours | aerobically |
|  | ATCC 15916 | Nutrient broth | 30 ℃, 26 hours | aerobically |
| *Pseudomonas brenneri* | ATCC 49642 | Nutrient broth | 30 ℃, 26 hours | aerobically |
| *Pseudomonas luteola* | ATCC 43273^T^ | BHI broth | 30 ℃, 22 hours | aerobically |
| *Pseudomonas oryzihabitans* | JCM 2952^T^ | BHI broth | 30 ℃, 22 hours | aerobically |
|  | JCM 3843 | Nutrient broth | 30 ℃, 22 hours | aerobically |
| *Pseudomonas pseudoalcaligenes* | JCM 5968^T^ | BHI broth | 26 ℃, 22 hours | aerobically |
| *Pseudomonas putida* | ATCC 12633^T^ | Nutrient broth | 30 ℃, 26 hours | aerobically |
|  | ATCC 49128 | Nutrient broth | 30 ℃, 22 hours | aerobically |
| *Pseudomonas stutzeri* | ATCC17588^T^ | BHI broth | 37 ℃, 22 hours | aerobically |
| *Pseudomonas tolaasii* | ATCC 33618^T^ | BHI broth | 26 ℃, 22 hours | aerobically |
| *Bacteroides vulgatus* | ATCC 8482^T^ | GAM broth ^D)^ | 37 ℃, 24 hours | anaerobically |
| *Bifidobacterium adolescentis* | ATCC15703^T^ | GAM broth | 37 ℃, 24 hours | anaerobically |
| *Blautia producta* | JCM 1471^T^ | GAM broth | 37 ℃, 18 hours | anaerobically |
| *Clostridium difficile* | DSM 1296^T^ | GAM broth | 37 ℃, 24 hours | anaerobically |
| *Clostridium perfringens* | JCM 1290^T^ | GAM broth | 37 ℃, 24 hours | anaerobically |
| *Collinsella aerofaciens* | DSM 3979^T^ | GAM broth | 37 ℃, 24 hours | anaerobically |
| *Enterococcus faecalis* | ATCC 19433^T^ | BHI broth | 37 ℃, 16 hours | aerobically |
| *Escherichia coli* | ATCC 11775^T^ | BHI broth | 37 ℃, 16 hours | aerobically |
| *Faecalibacterium prausnitzii* | ATCC 27768^T^ | GAM broth | 37 ℃, 72 hours | anaerobically |
| *Prevotella melaninogenica* | ATCC 25845^T^ | GAM broth | 37 ℃, 24 hours | anaerobically |
| *Lactobacillus brevis* | ATCC 14869^T^ | MRS broth ^E)^ | 37 ℃, 16 hours | anaerobically |
| *Lactobacillus casei* | ATCC 334^T^ | MRS broth | 37 ℃, 16 hours | anaerobically |
| *Lactobacillus fermentum* | ATCC 14931^T^ | MRS broth | 37 ℃, 16 hours | anaerobically |
| *Lactobacillus fructivorans* | ATCC 8288^T^ | MRS broth | 37 ℃, 16 hours | anaerobically |
| *Lactobacillus gasseri* | DSM 20243^T^ | MRS broth | 37 ℃, 16 hours | anaerobically |
| *Lactobacillus plantarum* | ATCC 14917^T^ | MRS broth | 37 ℃, 16 hours | anaerobically |
| *Lactobacillus reuteri* | JCM 1112^T^ | MRS broth | 37 ℃, 16 hours | anaerobically |
| *Lactobacillus ruminis* | JCM 1152^T^ | MRS broth | 37 ℃, 16 hours | anaerobically |
| *Lactobacillus sakei* | ATCC 15521^T^ | MRS broth | 37 ℃, 16 hours | anaerobically |
| *Staphylococcus aureus* | ATCC 12600^T^ | BHI broth | 37 ℃, 16 hours | aerobically |
| *Streptococcus mutans* | ATCC 27175^T^ | BHI broth | 37 ℃, 16 hours | aerobically |

^A^ BHI broth: Brain heart infusion broth (Beckton Dickinson Co.)

^B^ TS broth: Trypticase soy broth (Beckton Dickinson Co.)

^C^ Nutrient broth: (Nissui Pharmaceutical Co.)

^D^ GAM broth: Modified GAM broth (Nissui Pharmaceutical Co.) containing 1.0% (wt/vol) glucose (KANTO CHEMICAL CO., Tokyo, Japan)

^E^ MRS broth: (Beckton Dickinson Co., 288130)

Total bacterial cell counts of fresh cultures were determined using DAPI staining.

**Table S2. Designed primers for sequencing 23S rRNA**

| Primer | Sequence (5′-3′) | Annealing temperature (°C) | Reference |
| --- | --- | --- | --- |
| PLSU-30F | CGCATACGGTGGATGCCTTGGCAGT | 62 | This study |
| PLSU-470F | AGATAGGACGGAGCACGAGAAA | 55 | This study |
| PLSU-900F | AAGCTCGGAGATAGCTGGTTCTCC | 59 | This study |
| PLSU-1440F | TGCGCAACGTTAATCGACGCAGGGT | 61 | This study |
| PLSU-2060F | GGCGGCCGTAACTATAACGGTCCTA | 61 | This study |
| PLSU-2450F | GAGTAACGGAGGAGTACGAAGGTGC | 61 | This study |
| PLSU-470R | TTTCTCGTGCTCCGTCCTATCT | 55 | This study |
| PLSU-900R | GGAGAACCAGCTATCTCCGAGCTT | 59 | This study |
| PLSU-1440R | ACCCTGCGTCGATTAACGTTGCGCA | 61 | This study |
| PLSU-2060R | TAGGACCGTTATAGTTACGGCCGCC | 61 | This study |
| PLSU-2450R | GCACCTTCGTACTCCTCCGTTACTC | 61 | This study |
| PLSU-3000R | ACACACCCAACCTATCAACGTC | 55 | This study |
| PA-190R | TCGGTTGATTTCTTTTCCTCAGGG | 61 | This study |
| PA-220F | TWAGTAGTGGCGAGCGAACG | 60 | This study |
| PA-850R | AGCCGAAACAGTGCTCTACC | 60 | This study |
| PA-1000F | TGGTAAACGATGTGGGAAGGC | 60 | This study |
| PA-2070R | ATACACTGCATCTTCACAGCG | 58 | This study |
| PA-2630F | TGGACGTTTGAGATTTGAGAGGG | 60 | This study |

**Table S3. Newly acquired accession numbers**

| Taxon | Strain | Accession number |
| --- | --- | --- |
| *Pseudomonas aeruginosa* | ATCC 10145^T^ | LC515958 |
|  | ATCC 9027 | LC515959 |
|  | ATCC 15442 | LC515960 |
|  | JCM 2776 | LC515961 |
|  | JCM 5961 | LC515962 |
|  | DSM 6195 | LC515963 |
| *Pseudomonas alcaligenes* | ATCC 14909^T^ | LC515970 |
| *Pseudomonas fluorescens* | ATCC 13525^T^ | LC515971 |
| *Pseudomonas luteola* | ATCC 43273^T^ | LC515966 |
| *Pseudomonas oryzihabitans* | JCM 2952^T^ | LC515967 |
| *Pseudomonas pseudoalcaligenes* | JCM 5968^T^ | LC515964 |
| *Pseudomonas putida* | ATCC 12633^T^ | LC515968 |
| *Pseudomonas stutzeri* | ATCC17588^T^ | LC515965 |
| *Pseudomonas tolaasii* | ATCC 33618^T^ | LC515969 |

**Table S4. Partial 23S rRNA gene sequences of reference organisms with the s-Pa-F/s-Pa-R primer set**

| Taxon^A^ | Forward primer |  | Reverse primer |
| --- | --- | --- | --- |
|  | Sequence^B^ |  | Sequence^B^ |
| *P. aeruginosa* -specific primers | 5′ GTCGTCTTTTAGATGACGAAGTGG 3′ |  | 3′ACCATAGAAGCTGGTCGGTCT 5′ |
|  |  |  |  |
| Target site (*P. aeruginosa*) | 5′ GTCGTCTTTTAGATGACGAAGTGG 3′ |  | 5′ TGGTATCTTCGACCAGCCAGA 3′ |
| *Pseudomonas alcaligenes* | • •GTC• • • • •G • • ‐C •T • • • • • • • |  | • • • • • • • • • • • • • •G • •AT • • |
| *Pseudomonas fluorescens* | • •TA• • • • • • • • • • • • • • • • • • • • |  | • • • • • • • • • • • • • •G • •AT • • |
| *Pseudomonas luteola* | C• •T• • • •A • ‐ • •A • •G • • • • • • • |  | • • • • • • • • • • • • • •G • •AT • • |
| *Pseudomonas oryzihabitans* | TC •T• • • • • • • • •A •GA • • • • • • • |  | • • • • • • • • • • • • • •G • •AT • • |
| *Pseudomonas tolaasii* | •CTAA• • • • • • •T •AG • • • • • • • • |  | • • • • • • • • • • • • • •G • •AT • • |
| *Pseudomonas pseudoalcaligenes* | •CGT • • • • • • • • •ATG • • • • • • • • |  | • • • • • • • • • • • • • •G • •AT •G |
| *Pseudomonas putida* | • •T •C • ‐A • • • •GC • • • • • • • • • • |  | • • • • • • • • • • • • • •G • •AT •G |
| *Pseudomonas stutzeri* | •CTT • • • • • • • • •AAG• • • • • • • • |  | • • • • • • • • • • • • • •G • •AT •G |
| *Escherichia coli* | • ‐ ‐• ‐ •AC •AC •G • •CT • • • •CAA |  | • • • • • • • • • • • • •TGATTTC • |
| *Salmonella enterica* | • ‐ ‐• ‐ •AC •AC •G • •CT • • • •CAA |  | • • • • • • • • • • • • •TGA •TTC • |
| *Proteus mirabilis* | ‐ ‐A • ‐ •ACCAC •G • •CT • • • •CAA |  | • • • • • • • • • • • • •TG • •TT • • |
| *Citrobacter freundii* | • ‐ ‐• ‐ •AC •AC •G • •CT • • • • •AA |  | • • • • • • • • • • • • •TGATTTC • |
| *Buttiauxella agrestis* | • ‐ ‐• ‐ •AC •AC •G • •CT • • • • •AA |  | • • • • • • • • • • • • •TGC •TTC • |
| *Haemophilus influenzae* | • ‐ ‐• ‐ • •C •AC •GA •CT • • • • •AA |  | • • • • • • • • • • • • • •G •TTCA • |
| *Serratia marcescens* | ‐• ‐ ‐G •AC •AC •G • •CA • • • • •A • |  | • • • • • • • • • • • • •TG • •TTC • |
| *Shewanella frigidimarina* | • • ‐ ‐ ‐ •AC •AC •G• • •T • • • • •A • |  | • • • • • • • •G • • • •TGC •GTC • |
| *Shewanella algae* | • • • ‐ ‐ • ‐C •A• •GG • •T• • • • • • • |  | • • • • • • • •G • • • •TCC •GTC • |
| *Acinetobacter baumannii* | • ‐ •TGTAC • •GT •CAG • • • • • • • • |  | • • • •T • • •G • •G •TGT •GTC • |
| *Legionella pneumophila* | ‐C• ‐ ‐T • • •GGT •CAGA• • • • •CA |  | • • • •C • • •G • •G • •CT •TCC • |
| *Aeromonas hydrophila* | ‐A• ‐G •AC •AC •G • •CG• • • • CCA |  | • • • •CA • •G • • • •TCC •A •CT |
| *Moraxella catarrhalis* | • ‐ •CAG• • •ACTG ‐ • ‐ •A• • • • • • |  | • • • • • • • •G • • • •TGC •A •C • |

^A^ The positions of the target sites for the primers are as follows (numbering based on the *Pseudomonas aeruginosa* 23S rRNA gene sequence): s-Pa-F, nucleotides 1502 to 1525; s-Pa-R, nucleotides 1715 to 1735.

^B^ Only nucleotides that differ from nucleotides in the target sequences are shown.

**Table S5. Changes in fecal microbiota**

|  | Patient A | | | | | |  | Patient B | | | | |  | Patient C | | | |  | Patient D | | | | |
| --- | --- | --- | --- | --- | --- | --- | --- | --- | --- | --- | --- | --- | --- | --- | --- | --- | --- | --- | --- | --- | --- | --- | --- |
|  | Days of hospital stay | | | | | |  | Days of hospital stay | | | | |  | Days of hospital stay | | | |  | Days of hospital stay | | | | |
|  | 1 | 9 | 16 | 23 | 30 | 38 |  | 3 | 15 | 31 | 36 | 41 |  | 10 | 17 | 24 | 31 |  | 2 | 8 | 16 | 23 | 29 |
| Total bacterial counts | 7.5^A^ (100)^B^ | 9.9  (100) | 7.4  (100) | 9.3  (100) | 9.3  (100) | 9.6  (100) |  | 7.3  (100) | 7.8  (100) | 7.8  (100) | 7.5  (100) | 8.6  (100) |  | 8.2  (100) | 9.2  (100) | 11.1  (100) | 9.0  (100) |  | 7.6  (100) | 7.3  (100) | 9.1  (100) | 10.0  (100) | 8.6  (100) |
| Obligate anaerobe | 7.5  (89.6) | 9.9  (97.0) | 7.4  (97.3) | 9.0  (50.7) | 6.9  (0.4) | 9.2  (42.3) |  | 6.7  (26.6) | 6.5  (5.8) | 5.6  (0.6) | 5.8  (1.8) | 6.5  (0.7) |  | 7.9  (43.6) | 9.2  (86.1) | 11.1  (90.0) | 8.9  (94.5) |  | 7.6  (94.9) | 7.3  (99.3) | 7.8  (4.4) | 9.9  (97.0) | 8.6  (99.7) |
| *Clostridium coccoides* group | 7.0  (26.4) | 9.7  (61.0) | 6.9  (32.7) | 8.9  (42.4) | 6.3  (0.1) | 9.1  (30.0) |  | 5.1  (0.7) | 5.8  (1.1) | 5.1  (0.1) | <5.0  (0) | 5.8  (0.2) |  | 6.4  (1.6) | 8.4  (13.6) | 10.1  (8.3) | 7.6  (4.4) |  | 5.6  (1.1) | <5.0  (0) | 5.1  (0.0) | 9.1  (13.6) | 8.0  (20.5) |
| *C.leptum* subgroup | 7.2  (40.8) | 9.5  (35.5) | 5.9  (3.8) | 7.9  (3.8) | <5.0  (0) | 8.6  (11.4) |  | 6.0  (4.8) | 5.8  (1.1) | <5.0  (0) | 5.1  (0.4) | 5.1  (0.0) |  | 6.7  (2.6) | 8.8  (36.0) | 10.7  (34.0) | 7.8  (7.3) |  | 6.3  (5.1) | 5.8  (3.2) | 5.3  (0.0) | 9.4  (25.4) | 8.1  (26.5) |
| *Bacteroides fragilis* group | <5.0  (0) | <5.0  (0) | <5.0  (0) | <5.0  (0) | <5.0  (0) | <5.0  (0) |  | 6.5  (15.1) | 6.2  (2.6) | 5.5  (0.5) | 5.0  (0.3) | 6.1  (0.3) |  | 6.6  (2.5) | 8.6  (26.5) | 10.8  (42.8) | 7.5  (3.6) |  | 5.2  (0.4) | <5.0  (0) | <5.0  (0) | 9.4  (25.7) | 7.9  (17.4) |
| *Bifidobacterium* | 6.1  (3.6) | 6.3  (0.0) | <5.0  (0) | <5.0  (0) | 6.0  (0.0) | 5.5  (0.0) |  | 5.8  (3.0) | 5.6  (0.6) | <5.0  (0) | 5.2  (0.5) | 5.8  (0.2) |  | 6.8  (3.3) | 7.8  (4.1) | 9.7  (3.4) | 8.0  (11.0) |  | 6.2  (4.6) | 7.1  (55.7) | 7.8  (4.4) | 8.8  (6.5) | 8.1  (28.0) |
| *Atopobium* cluster | 6.6  (12.7) | 7.7  (0.5) | 7.2  (60.8) | 8.0  (4.5) | 6.7  (0.3) | 7.5  (0.9) |  | 5.5  (1.7) | 5.3  (0.4) | <5.0  (0) | 5.3  (0.6) | 5.3  (0.0) |  | 7.0  (5.6) | 8.0  (5.8) | 9.3  (1.5) | 7.6  (4.4) |  | 6.4  (7.0) | 6.3  (9.4) | 5.0  (0.0) | 8.7  (5.9) | 7.5  (7.3) |
| *Prevotella* | 6.3  (6.1) | <5.0  (0) | <5.0  (0) | <5.0  (0) | <5.0  (0) | <5.0  (0) |  | 5.4  (1.3) | <5.0  (0) | <5.0  (0) | <5.0  (0) | <5.0  (0) |  | 7.7  (28.0) | 6.0  (0.1) | <5.0  (0) | 8.8  (63.8) |  | 7.5  (76.7) | 6.8  (31.0) | <5.0  (0) | 9.3  (19.9) | 5.3  (0.0) |
| Faculative anaerobes and aerobes | 6.6  (10.4) | 8.4  (3.0) | 5.8  (2.7) | 9.0  (49.3) | 9.3  (99.6) | 9.3  (57.7) |  | 7.1  (73.4) | 7.8  (94.2) | 7.8  (99.4) | 7.5  (98.2) | 8.6  (99.3) |  | 8.0  (56.4) | 8.4  (13.9) | 10.2  (10.0) | 7.7  (5.5) |  | 6.3  (5.1) | 5.1  (0.7) | 9.1  (95.6) | 8.4  (3.0) | 6.1  (0.3) |
| Total lactobacilli  (formerly total *Lactobacillus*) | <3.9  (0) | 6.8  (0.1) | 4.6  (0) | 8.5  (14.1) | 8.8  (32.0) | 9.1  (30.3) |  | 5.4  (1.4) | 5.2  (0.2) | 6.7  (8.1) | 5.9  (2.8) | 5.7  (0.1) |  | 7.8  (36.0) | 7.0  (0.6) | 8.3  (0.1) | 7.5  (3.6) |  | 4.5  (0.1) | 4.9  (0.5) | 7.0  (0.8) | 7.7  (0.5) | 5.2  (0.0) |
| *Enterobacteriaceae* | 6.6  (10.4) | 5.8  (0.0) | 4.3  (0) | 8.4  (12.3) | 8.8  (31.6) | 9.0  (24.8) |  | 5.1  (0.6) | 7.0  (15.7) | 6.3  (3.0) | 6.6  (13.6) | 7.0  (2.6) |  | 7.3  (12.2) | 6.0  (0.1) | 4.2  (0.0) | 5.3  (0.0) |  | 5.6  (1.1) | <4.0  (0) | 7.3  (1.6) | 7.9  (0.9) | <4.0  (0) |
| *Enterococcus* | 4.0  (0.0) | 4.2  (0.0) | <3.9  (0) | 8.7  (22.8) | 7.1  (0.6) | 7.0  (0.3) |  | 7.1  (71.4) | <3.9  (0) | 7.8  (86.9) | 7.4  (81.5) | 8.5  (82.3) |  | 6.3  (1.2) | 8.3  (13.0) | 10.2  (9.9) | 7.3  (1.9) |  | 6.1  (3.7) | 4.5  (0.1) | 9.1  (93.1) | 8.2  (1.6) | 6.0  (0.2) |
| *Staphylococcus* | 4.0  (0.0) | 8.4  (2.9) | 5.7  (2.4) | 6.0  (0.1) | 5.6  (0.0) | 6.0  (0.0) |  | <2.9  (0.0) | <2.9  (0) | <2.9  (0) | <2.9  (0) | <2.9  (0) |  | 7.0  (5.4) | 4.9  (0.0) | 6.3  (0.0) | 5.3  (0.0) |  | 3.8  (0.0) | 4.5  (0.1) | 6.1  (0.1) | 5.8  (0.0) | 4.0  (0.0) |
| *Pseudomonas aeruginosa* | <2.3  (0) | <2.3  (0) | <2.3  (0) | <2.3  (0) | 8.9  (35.4) | 7.9  (2.3) |  | 2.7  (0.0) | 7.7  (78.3) | 6.0  (1.4) | 5.0  (0.3) | 7.8  (14.3) |  | 6.4  (1.6) | 6.4  (0.2) | 5.8  (0.0) | <2.3  (0) |  | 4.9  (0.2) | 2.7  (0.0) | 5.6  (0.0) | <2.3  (0) | <2.3  (0) |

^A^ Log_10_ cells/g of feces

^B^ Composition ratio (%)
